# Time trends in the incidence rates of venous thromboembolism following colorectal resection by indication and operative technique

**DOI:** 10.1111/codi.16233

**Published:** 2022-07-19

**Authors:** Christopher A. Lewis‐Lloyd, Colin J. Crooks, Joe West, Oliver Peacock, David J. Humes

**Affiliations:** ^1^ Gastrointestinal Surgery, National Institute for Health Research (NIHR) Nottingham Biomedical Research Centre (BRC), School of Medicine, Queen's Medical Centre Nottingham University Hospitals NHS Trust and the University of Nottingham Nottingham UK; ^2^ Gastrointestinal and Liver Theme, National Institute for Health Research (NIHR) Nottingham Biomedical Research Centre (BRC), School of Medicine, Queen's Medical Centre) Nottingham University Hospitals NHS Trust and the University of Nottingham Nottingham UK; ^3^ Population and Lifespan Sciences University of Nottingham, School of Medicine Nottingham UK; ^4^ Department of Colon and Rectal Surgery, MD Anderson Cancer Center University of Texas Houston Texas USA

**Keywords:** colorectal surgery, epidemiology, general surgery, pulmonary embolism, venous thromboembolism, venous thrombosis

## Abstract

**Aim:**

It is important for patient safety to assess if international changes in perioperative care, such as the focus on venous thromboembolism (VTE) prevention and minimally invasive surgery, have reduced the high post colectomy VTE risks previously reported. This study assesses the impact of changes in perioperative care on VTE risk following colorectal resection.

**Method:**

This was a population‐based cohort study of colectomy patients in England between 2000 and 2019 using a national database of linked primary (Clinical Practice Research Datalink) and secondary (Hospital Episode Statistics) care data. Within 30 days following colectomy, absolute VTE rates per 1000 person‐years and adjusted incidence rate ratios (aIRRs) using Poisson regression for the per year change in VTE risk were calculated.

**Results:**

Of 183 791 patients, 1337 (0.73%) developed 30‐day postoperative VTE. Overall, VTE rates reduced over the 20‐year study period following elective (relative risk reduction 31.25%, 95% CI 5.69%–49.88%) but not emergency surgery. Similarly, yearly changes in VTE risk reduced following minimally invasive resections (elective benign, aIRR 0.93, 95% CI 0.90–0.97; elective malignant, aIRR 0.94, 95% CI 0.91–0.98; and emergency benign, aIRR 0.96, 95% CI 0.92–1.00) but not following open resections. There was a per year VTE risk increase following open emergency malignant resections (aIRR 1.02, 95% CI 1.00–1.04).

**Conclusion:**

Yearly VTE risks reduced following minimally invasive surgeries in the elective setting yet in contrast were static following open elective colectomies, and following emergency malignant resections increased by almost 2% per year. To reduce VTE risk, further efforts are required to implement advances in surgical care for those having emergency and/or open surgery.

**What does this paper add to the literature?**

Perioperative care has changed with an increase in venous thromboembolism (VTE) prevention. VTE rates have reduced following minimally invasive but not open colectomies by 4%–7% annually. However, rates post emergency malignant resections have increased by almost 2% annually, suggesting future focus on improving postoperative emergency colectomy VTE rates is needed.

## INTRODUCTION

Colorectal resection, the second most common major abdominal operation in the developed world, forms a substantial proportion of resectional abdominal surgery [1]. In the United States (US) alone, approximately 320 000 colectomies are performed annually [2]. Venous thromboembolism (VTE) is a potentially preventable, fatal complication following colectomy occurring in 1.92% of patients within 30 days following colorectal cancer (CRC) resection [3].

Within the last two decades colorectal surgical care has changed. Globally VTE became a significant postoperative complication with multiple high‐quality studies devoted to investigating methods of reducing its risk following colorectal resection [4, 5, 6, 7]. In response, the American College of Chest Physicians in 2008 [8] and the National Institute for Health and Care Excellence (NICE) in 2010 [9] published landmark guidance on VTE prevention following abdominal and pelvic surgery that has been adopted internationally [10, 11, 12, 13, 14]. This guidance advocated the use of inpatient combined pharmacological and mechanical VTE prophylaxis following gastrointestinal operations for benign disease and specifically extended postoperative pharmacological prophylaxis following cancer resections for 28 days [15, 16]. VTE is considered so important that it forms part of the World Health Organization's (WHO) Surgical Safety Checklist and is a performance measure in the US, United Kingdom (UK) and Australia with emphasis on timely risk assessment and appropriate prophylaxis prescription [17, 18]. Furthermore, the impact of incidental VTE is becoming apparent, with evidence suggesting similar rates of morbidity and mortality as those with symptomatic VTE in cancer patients and the reduction of asymptomatic VTE observed in laparoscopic CRC surgery with extended VTE prophylaxis use [4, 19].

In addition to VTE guidance, minimally invasive resection has been increasingly adopted for benign and malignant colorectal disease in developed and developing nations globally [20, 21, 22, 23, 24]. Minimally invasive surgery is coupled with the increased global utilization of elective enhanced recovery after surgery (ERAS) programmes that have reduced perioperative complications and hospital length of stay [25, 26]. The current literature debates the effect that minimally invasive surgery has on VTE risk [27, 28, 29, 30]. Reports state that prolonged operating times and iatrogenic pneumoperitoneum increase venous stasis, yet population‐based evidence from literature review suggests minimally invasive compared to open colectomy reduces VTE risk [31, 32, 33]. However, none of these studies reports VTE risk by operative technique stratified by admission type, examines incidence rates or accounts for time within their analyses.

The focus on VTE prevention, changes in operative techniques and ERAS will probably have altered VTE risk following colectomy, yet this has not been established. This study aims to quantify variations in VTE rates over the last 20 years in relation to international changes in perioperative care following colorectal resection.

## METHODS

The study was approved by the Independent Scientific Advisory Committee approval board (Protocol 19_180RA3).

### Data sources

This study utilized three validated and linked healthcare databases that have been described previously [34, 35, 36]. The Clinical Practice Research Datalink (CPRD), comprising the CPRD GOLD and Aurum databases, contains primary care prescription and diagnostic data for approximately 60 million patients of the UK general population, with 16 million actively contributing data [37]. Hospital Episode Statistics (HES) since 1989 has compiled detailed records for each episode of admitted patient care delivered in England, either by the National Health Service (NHS) or commissioned by the NHS within the independent sector [38]. HES patient records are coded using a combination of the International Statistical Classification of Diseases and Related Health Problems 10th Revision (ICD‐10) codes for discharge diagnoses and the Office of Population, Censuses and Surveys Classification of Surgical Operations and Procedures version 4 (OPCS‐4) codes for detailing procedures relating to an admission. These data have been shown to be demographically equivalent to data from the UK population having been compared to Office for National Statistics data [39, 40].

### Cohort

The cohort of patients undergoing colorectal resections between the years 2000 and 2019 was identified using OPCS‐4 codes (Appendix S1) from HES data that were linked to CPRD GOLD and Aurum general practitioner practices. Within the CPRD databases, data quality can be assessed using two sets of metrics, acceptability and the up to standard date. Data are only considered acceptable if specific quality measures are met including valid age, gender, accuracy of recorded patient events and registration status. The up to standard date focuses on the accuracy of data continuity recorded by individual primary care practices for patients and is the latest date at which these practices met the minimum quality criteria outlined by the CPRD [41]. Therefore, patients were only included within the validated cohort if data collection time periods for both primary and secondary care databases coincided and their data were classed as up to research standard and acceptable [42].

Completely endoscopic operations and those confined to the anal canal were excluded, including patients aged <18 years old at the date of operation. Patients identified as having a VTE event prior to resection were also excluded due to their inherently increased risk of VTE [43]. Previous VTE was defined using the same definition of VTE for the entire study. Patient follow‐up included VTEs on the day of surgery, and lasted until earliest date of a VTE event, death, change to a non‐participating general practice, or for 30 days after surgery.

### Exposures

Calendar year was the main exposure of interest, defined as a continuous variable and as a binary variable pre‐ and post‐2010 (the year NICE guidance [CG92] from England advocating extended VTE prophylaxis in CRC resections was published). Other exposures were selected from previous literature as follows [44, 45]. Surgical indication was defined as malignant or benign. A malignant indication was defined within the CPRD and HES data using relevant ICD‐10 codes for CRC (C18–C20, excluding C18.1). A benign indication was defined using ICD‐10 discharge codes, including inflammatory bowel disease, diverticular disease and other (Appendix S1). Admission type was defined as elective or emergency based on the admission classification recorded for the surgical procedure in HES data. Age was categorized into <60, 60–69, 70–79 and ≥80 years. Sex was defined from HES data as either male or female. Comorbidity was classified using the Charlson index prior to surgical admission and determined from CPRD and HES data; categories used were 0, 1 and ≥2 [46]. Ethnicity was determined from HES and categorized as white, other or unknown. Operative technique was classified into open, minimally invasive or those converted from minimally invasive to open surgery. Minimally invasive technique was defined as laparoscopic (Y50.8, Y57.1 and Y75.2) or robotic (Y75.3) surgery using OPCS‐4 codes. Hospital length of stay was defined from HES as the difference between colectomy admission episode start and end dates.

National NICE guidance from England on postoperative VTE prevention, published in 2010, stated that those undergoing abdominal surgery at risk of VTE should receive combined mechanical and chemical VTE prophylaxis during admission and those undergoing cancer surgery should receive an extended 28 days of postoperative VTE chemoprophylaxis [15]. Therefore, within a population‐based cohort from a nationally organized health service, all patients prior to the year 2010 were considered to have had inpatient VTE prophylaxis. However, from the year 2010 onwards those undergoing benign resections were deemed to have received inpatient but not extended or post‐discharge VTE prophylaxis while those undergoing malignant resections were deemed to have received inpatient and a total of 28 days extended VTE prophylaxis and, where applicable, prophylaxis into the post‐discharge period.

### Outcomes

The primary outcome was a VTE event diagnosis, either a pulmonary embolism or a peripheral deep vein thrombosis, defined from medical and ICD‐10 codes in the linked CPRD and HES datasets 30 days post resection. VTE events were only considered valid if supported by either a prescription for any anticoagulant medication from the British National Formula licensed to treat a VTE in the UK or other evidence of treatment within an anticoagulation clinic (e.g., a medical code) between 15 days before and 90 days after VTE diagnosis, or a date of death within 30 days of the event. This time frame was used to allow for anticoagulant prescribing in the community to follow on from anticoagulation clinic or inpatient prescribing for a VTE event. Furthermore, VTE as an underlying cause of death was included as evidence of VTE diagnosis. A 30‐day postoperative follow‐up was selected because this is the period of time required for the intervention of extended VTE prophylaxis and is a standard follow‐up end‐point in other national population‐based cohort studies, allowing ease of comparison [3]. Only the first confirmed VTE episode was incorporated within the analysis. This definition using primary care data has been formally validated and published, specifically validating a VTE diagnosis against individual patient records with a positive predictive value of 84% [47] and used in the authors' previous work [44].

### Statistical analysis

Cohort demographics were presented as proportions and stratified by admission type and surgical indication. 30‐day post colectomy incidence risks, presented as percentages, and absolute VTE incidence rates (IR) per 1000 person‐years were calculated for each year of the study period and stratified a priori by admission type and surgical indication [15, 16, 44].

Poisson regression was used to calculate adjusted incidence rate ratios (aIRRs) for linear trend in year‐on‐year VTE risk, adjusted for all other exposures within the analyses and where relevant the relative change in VTE risk as a percentage over the 20‐year study period. Effect modification, significance set at *P* < 0.1 using a generalized likelihood ratio test, was assessed within the multivariable model to test for any interactions between linear trend by year of colectomy and admission type, surgical indication and operative technique.

In addition, a sensitivity analysis examining whether there was an interaction between the linear trend in VTE rates by year and pre‐ and post‐2010, the year NICE guidance on extended VTE prophylaxis use post malignant colectomy was published, was conducted.

All data management and analyses were performed using Stata SE^®^ version 16.1 (StataCorp LLC).

## RESULTS

### Cohort demographics

Overall, 183 791 patients in the validated cohort underwent colorectal resection and 1337 (0.73%) developed VTE within 30 days after surgery with an absolute IR of 90.67 (95% CI 85.94–95.66) per 1000 person‐years. Most patients were of white ethnicity (91.19%) and 93 598 (50.93%) were male.

There was an increase in minimally invasive surgery over time. Between 2000 and 2019 the yearly proportion of colectomies performed minimally invasively increased from 0.59% to 73.31% in the elective setting and from 0.36% to 26.17% in the emergency setting (both *P* < 0.0001, *χ* ^2^ test for trend; see Figure S1). Overall hospital length of stay decreased over the study period from 12 (interquartile range [IQR] 9–18) to 6 (IQR 4–11) days (*P* < 0.0001, Kruskal–Wallis test; see Table 1).

**TABLE 1:** Demographics of colorectal resection cohort, by admission type and indication for surgery

	Number 183 791			
	Emergency (no. 62 183)		Elective (no. 121 608)	
	benign (no. = 41 095)	malignant (no. = 21 088)	benign (no. = 36 183)	malignant (no. = 85 425)
	No. (%)	No. (%)	No. (%)	No. (%)
Age (years)				
<60	19 319 (47.01)	4537 (21.51)	19 439 (53.72)	17 951 (21.01)
60–69	7448 (18.12)	4542 (21.54)	7736 (21.38)	23 413 (27.41)
70–79	8383 (20.40)	6266 (29.71)	6598 (18.24)	27 936 (32.70)
≥80	5945 (14.47)	5743 (27.23)	2410 (6.66)	16 125 (18.88)
Sex				
Male	18 859 (45.89)	10 481 (49.70)	15 493 (42.82)	48 765 (57.09)
Female	22 236 (54.11)	10 607 (50.30)	20 690 (57.18)	36 660 (42.91)
Charlson score				
0	17 547 (42.70)	6380 (30.25)	15 422 (42.62)	6678 (7.82)
1	3389 (8.25)	2777 (13.17)	3435 (9.49)	7367 (8.62)
≥2	20 159 (49.05)	11 931 (56.58)	17 326 (47.88)	71 380 (83.56)
Ethnicity				
White	37 018 (90.08)	18 883 (89.54)	33 190 (91.73)	78 511 (91.91)
Other	2452 (5.97)	955 (4.53)	2166 (5.99)	3982 (4.66)
Unknown	1625 (3.95)	1250 (5.93)	827 (2.29)	2932 (3.43)
Operative technique				
Open	36 098 (87.84)	19 239 (91.23)	23 583 (65.18)	50 028 (58.56)
Minimally invasive converted to open	1927 (4.69)	535 (2.54)	2042 (5.64)	5053 (5.92)
Minimally Invasive	3070 (7.47)	1314 (6.23)	10 558 (29.18)	30 344 (35.52)
VTE at 30 days				
Yes	380 (0.92)	295 (1.40)	169 (0.47)	493 (0.58)
No	40 715 (99.08)	20 793 (98.60)	36 014 (99.53)	84 932 (99.42)

*Note:* Minimally invasive surgery consists of procedure completed by either a laparoscopic or robotic technique.

Abbreviation: VTE, venous thromboembolism.

### Venous thromboembolism following colorectal resection over time

#### Thirty‐day rates and risks of VTE per year by admission type

**FIGURE 1:**
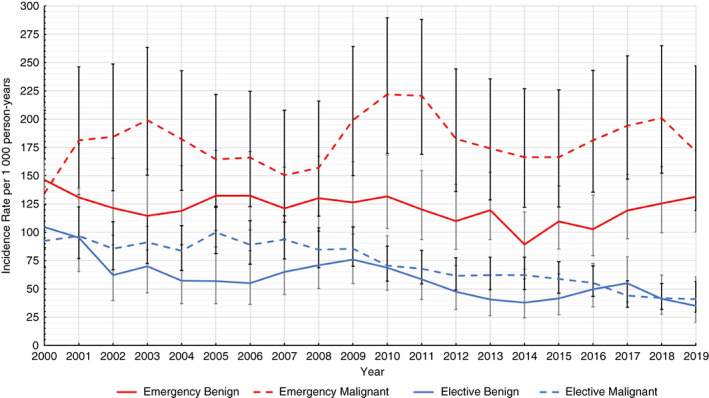
Three‐year rolling averaged 30‐day venous thromboembolism rates following colorectal resection stratified by admission type and surgical indication. Error bars indicate 95% confidence intervals.

Patients undergoing elective colorectal resections had a 30‐day VTE IR of 66.34 (95% CI 61.47–71.59) per 1000 person‐years (0.54%) and overall significant relative VTE risk reduction of 31.25% (95% CI 5.69%–49.88%) over the 2000–2019 study period (year‐on‐year VTE risk change, aIRR, 0.98, 95% CI 0.97–1.00; see Table 2), with yearly reductions in VTE rates observed prior to 2010 (see Figure 1). However, no statistically significant relative VTE risk change was detected in those undergoing emergency resections over the 20‐year study period (relative VTE risk increase 22.38%, 95% CI −7.39%–61.73%; year‐on‐year VTE risk change, aIRR 1.01, 95% CI 1.00–1.02), with a 30‐day VTE IR of 141.60 (95% CI 131.31–152.70) per 1000 person‐years (1.09%) (see Figure 1 and Table 3).

**TABLE 2:** Absolute rates of venous thromboembolism 30 days after elective surgery

Elective (no. 121 608)					
	Event no.	Person‐years	Rate per 1000 person‐years (95% CI)	Unadjusted incidence rate ratio (95% CI)	Adjusted incidence rate ratio^a^ (95% CI)
Surgical indication					
Benign	169	2.97	56.81 (48.86–66.06)	1.00 (Reference)	1.00 (Reference)
Malignant	493	7.00	70.38 (64.44–76.88)	1.24 (1.04–1.48)	0.96 (0.79–1.16)
Age (years)					
<60	122	3.10	39.34 (32.94–46.98)	1.00 (Reference)	1.00 (Reference)
60–69	170	2.57	66.22 (56.98–76.96)	1.68 (1.33–2.12)	1.57 (1.23–1.99)
70–79	255	2.82	90.52 (80.07–102.34)	2.30 (1.85–2.86)	2.09 (1.67–2.63)
≥80	115	1.49	76.98 (64.12–92.41)	1.96 (1.52–2.52)	1.78 (1.36–2.32)
Sex					
Male	375	5.25	71.37 (64.50–78.98)	1.00 (Reference)	1.00 (Reference)
Female	287	4.73	60.74 (54.10–68.19)	0.85 (0.73–0.99)	0.86 (0.74–1.01)
Charlson score					
0	88	1.82	48.22 (39.13–59.43)	1.00 (Reference)	1.00 (Reference)
1	62	0.89	69.88 (54.48–89.63)	1.45 (1.05–2.01)	1.21 (0.86–1.69)
≥2	512	7.27	70.45 (64.61–76.83)	1.46 (1.17–1.83)	1.27 (0.99–1.62)
Ethnicity					
White	612	9.17	66.73 (61.65–72.23)	1.00 (Reference)	1.00 (Reference)
Other	19	0.51	37.50 (23.92–58.80)	0.56 (0.36–0.89)	0.70 (0.44–1.10)
Unknown	31	0.30	102.95 (72.40–146.38)	1.54 (1.08–2.21)	1.40 (0.97–2.01)
Operative technique					
Open	498	6.01	82.80 (75.84–90.40)	1.00 (Reference)	1.00 (Reference)
Minimally invasive converted to open	33	0.58	56.69 (40.30–79.74)	0.68 (0.48–0.97)	0.75 (0.52–1.08)
Minimally invasive	131	3.38	38.73 (32.63–45.96)	0.47 (0.39–0.57)	0.53 (0.43–0.66)
Year (2000–2019)					
Per year change	–	–	–	0.96 (0.94–0.97)	0.98 (0.97–1.00)

*Note*: Minimally invasive surgery consists of procedure completed by either a laparoscopic or robotic technique.

^a^ Adjusted for surgical indication, age, sex, Charlson score, ethnicity, operative technique and year.

**TABLE 3:** Absolute rates of venous thromboembolism 30 days after emergency surgery

	Emergency (no. 62 183)				
	Event no.	Person‐years	Rate per 1000 person‐years (95% CI)	Unadjusted incidence rate ratio (95% CI)	Adjusted incidence rate ratio^a^ (95% CI)
Surgical indication					
Benign	380	3.14	121.20 (109.61–134.02)	1.00 (Reference)	1.00 (Reference)
Malignant	295	1.63	180.80 (161.30–202.66)	1.49 (1.28–1.74)	1.24 (1.05–1.45)
Age (years)					
<60	157	1.94	81.08 (69.34–94.80)	1.00 (Reference)	1.00 (Reference)
60–69	148	0.93	159.33 (135.62–187.18)	1.97 (1.57–2.46)	1.73 (1.38–2.18)
70–79	201	1.09	184.65 (160.81–212.02)	2.28 (1.85–2.81)	1.94 (1.56–2.42)
≥80	169	0.81	207.86 (178.77–241.68)	2.56 (2.06–3.19)	2.12 (1.68–2.69)
Sex					
Male	301	2.27	132.78 (118.59–148.66)	1.00 (Reference)	1.00 (Reference)
Female	374	2.50	149.60 (135.18–165.56)	1.13 (0.97–1.31)	1.09 (0.93–1.26)
Charlson score					
0	209	1.89	110.30 (96.31–126.31)	1.00 (Reference)	1.00 (Reference)
1	74	0.48	154.82 (123.27–194.43)	1.40 (1.08–1.83)	1.12 (0.86–1.47)
≥2	392	2.39	163.74 (148.31–180.78)	1.48 (1.26–1.76)	1.17 (0.98–1.39)
Ethnicity					
White	616	4.30	143.21 (132.34–154.98)	1.00 (Reference)	1.00 (Reference)
Other	21	0.27	77.34 (50.42–118.61)	0.54 (0.35–0.83)	0.67 (0.43–1.04)
Unknown	38	0.19	195.85 (142.51–269.16)	1.37 (0.99–1.90)	1.32 (0.94–1.83)
Operative technique					
Open	631	4.21	149.88 (138.63–162.04)	1.00 (Reference)	1.00 (Reference)
Minimally invasive converted to open	16	0.20	80.40 (49.26–131.24)	0.54 (0.33–0.88)	0.63 (0.38–1.04)
Minimally invasive	28	0.36	78.25 (54.03–113.34)	0.52 (0.36–0.76)	0.58 (0.40–0.86)
Year (2000–2019)					
Per year change	–	–	–	1.00 (0.98–1.01)	1.01 (1.00–1.02)

*Note:* Minimally invasive surgery consists of procedure completed by either a laparoscopic or robotic technique.

^a^ Adjusted for surgical indication, age, sex, Charlson score, ethnicity, operative technique and year.

#### Thirty‐day VTE risks per year by operative technique

Minimally invasive compared to open surgery reduced VTE risk by approximately 50% following both elective and emergency colorectal resections (aIRR 0.53, 95% CI 0.43–0.66; and aIRR 0.58, 95% CI 0.40–0.86 respectively). There were also significant interactions between the linear trend in VTE risk and both admission type, *P* = 0.0055, and operative technique, *P* = 0.0084. Therefore, for the remaining results the linear trend in VTE risks were further stratified by operative technique as in Table S1. For the final multivariable analysis, procedures that began using a minimally invasive approach were classed as minimally invasive surgery due to the overall small numbers and reduced statistical power within the minimally invasive converted to open subgroup.

##### Elective resections

Following elective benign and malignant minimally invasive colorectal resections a 6.78% and 5.62% year‐on‐year reduction in VTE risk was observed (aIRR 0.93, 95% CI 0.90–0.97; and aIRR 0.94, 95% CI 0.91–0.98, respectively) with 30‐day VTE IR per 1000 person‐years of 32.57 (95% CI 23.27–45.58; 0.27%) and IR 44.51 (95% CI 37.48–52.86; 0.37%) respectively. Following elective benign or malignant open resections no significant year‐on‐year VTE risk reduction was observed (aIRR 0.98, 95% CI 0.96–1.00, and aIRR 0.99, 95% CI 0.98–1.01 respectively) with 30‐day VTE IR per 1000 person‐years of 69.92 (95% CI 59.07–82.77; 0.57%) and IR 88.88 (95% CI 80.19–98.51; 0.73%) respectively.

##### Emergency resections

In the emergency setting, patients undergoing minimally invasive benign colorectal resections had a significant year‐on‐year reduction in VTE risk of 4.24% (aIRR 0.96, 95% CI 0.92–1.00) with a 30‐day VTE IR of 58.89 (95% CI 39.47–87.86) per 1000 person‐years (0.48%). No significant year‐on‐year reduction in VTE risk was observed in emergency malignant minimally invasive resections (aIRR 0.97, 95% CI 0.93–1.01) with a 30‐day VTE IR of 133.98 (95% CI 86.44–207.66) per 1000 person‐years (1.08%). In patients undergoing emergency open colorectal surgery, those undergoing cancer resection had a significant 1.89% year‐on‐year increase in VTE risk (aIRR 1.02, 95% CI 1.00–1.04) with a 30‐day VTE IR of 185.52 (95% CI 164.84–208.79) per 1000 person‐years (1.43%). In benign emergency open resections, no significant year‐on‐year increase in VTE risk was seen (aIRR 1.01, 95% CI 0.99–1.02) with a 30‐day VTE IR of 130.51 (95% CI 117.63–144.80) per 1000 person‐years (0.99%).

#### Postoperative VTE rates pre‐ and post‐2010

Within the final model, no significant effect modification, *P* = 0.9305, was found between year‐on‐year VTE trend post colorectal resection and pre/post the year 2010, the year NICE guidance on extended VTE prophylaxis use was published, with no overall significant step change in VTE risk between pre‐ and post‐2010 observed (aIRR 0.96, 95% CI 0.78–1.19). No change in effect modification or step change in VTE risk was observed using 1‐ or 2‐year washout periods post the introduction of NICE guidelines. This was despite the overall reduction in VTE rates seen in elective colorectal resections that began prior to the year 2010 (see Figure 1).

## DISCUSSION

### Overview

This population‐based cohort describes an overall relative risk reduction of 31.25% of 30‐day VTE following elective colorectal resections over the last 20 years, with no overall relative change in VTE risk after emergency resections. This decrease in VTE risk appears partially attributable to the increasing utilization of minimally invasive surgery approximately halving the risk compared to open surgery. This is potentially due to improvements in perioperative surgical care, such as the associated use of ERAS programmes and continued focus on VTE prevention seen in this patient subgroup. This increased focus on VTE prevention through the WHO Surgical Safety Checklist and publication of guidelines on VTE prevention and extended VTE prophylaxis, although not independently affecting VTE risk, has probably in part contributed to the overall reduction in VTE rates observed. However, other factors around advancements in perioperative care seem to be important in VTE prevention. Therefore, adoption of advances in perioperative care that have occurred in elective and or minimally invasive colectomy is imperative in patients undergoing open colorectal surgery, especially in the emergency setting for malignant disease, if we are to deliver further reductions in VTE in this high‐risk population.

### Strengths and limitations

The main strength of our large population study is the power to stratify the observed VTE time trends by type of admission, indication and operation. By using an unselected population‐based cohort study we were able to present estimates that will be generalizable to other populations with universal coverage and developed healthcare systems. A further advantage of our study is that the VTE definition has previously been validated, and it captures outpatient VTEs from both primary as well as those admitted in secondary care. This thereby reduces the surveillance bias that can occur if patients are solely identified whilst hospitalized [47, 48].

A limitation of the routine data is that we were unable to assess the effect of VTE prophylaxis directly, as hospital prescribing at patient level is not currently available from NHS Digital. However, recent national audit data report 95%–96% of NHS acute admissions were risk assessed for VTE and therefore for the appropriateness of VTE prophylaxis [49, 50]. This does not affect the interpretation of our results unduly as it is expected that all patient subgroups in this cohort will have prophylaxis prescribed that reflects the secular trends within the country, although this could potentially lead to misclassification bias of those receiving extended VTE prophylaxis. Clearly, on an individual level, whether or not prophylaxis was prescribed and for how long is very important in terms of individual risk but the overview of year‐on‐year change in VTE risk following resection is not otherwise possible due to the need for very large numbers of patients to analyse. As with any population‐based database, there are always concerns surrounding the accuracy of recorded data. However, the databases utilized within this study have been extensively used, previously validated and contain built‐in metrics to assure data quality and accuracy, including the use of a validated definition of a VTE outcome [35, 41, 44, 47].

### Elective colorectal resection VTE risks

Following elective surgery, we report annual reductions in VTE risk post minimally invasive colorectal resections. This contrasts with previous data showing no change or an increase in annual VTE risk post overall and elective malignant colectomy [51, 52, 53, 54]. However, none of these studies reported yearly VTE rates by admission type and operative technique. Ghadban et al. [51] solely assessed in‐hospital VTE events and Yhim et al. [54] examined a study interval predominantly encompassing years prior to 2010.

Interestingly, both elective benign and malignant minimally invasive VTE rates decreased year‐on‐year during the study even though 28‐day extended VTE prophylaxis was only recommended following the malignant resections [15, 16, 55]. This may be related to a lower base line risk of VTE in those with benign disease compared to those with malignant disease. Furthermore, the widespread introduction of minimally invasive surgery and other perioperative care improvements that are associated with it might have overshadowed the protective effect of extended VTE prophylaxis.

Approximately two‐thirds of colorectal resections were performed minimally invasively by the end of the study, in contrast to less than 1% at its start, in keeping with contemporary National Bowel Cancer Audit data [20]. There is dispute surrounding the impact of minimally invasive colorectal resection on VTE risk as there will be a selection bias of lower risk patients being selected for this surgery. However, this selection bias may be limited as by the end of the study approximately three‐quarters of patients underwent a minimally invasive procedure. Overall, meta‐analysis and population‐based data support our study's finding that VTE risk is reduced post minimally invasive colectomy [27, 29, 30, 56, 57, 58]. The low absolute VTE rates and yearly reduction in VTE risk in elective malignant minimally invasive resections may now suggest that this subgroup no longer requires extended VTE prophylaxis. The cost‐effectiveness of extended VTE prophylaxis is debated within the literature and is particularly important within public health systems such as the NHS [59, 60]. However, the low rates observed may be partially related to the current use of extended VTE prophylaxis in CRC resectional patients and its benefit has been demonstrated in randomized controlled trials [4].

Alongside the increase in minimally invasive resections was the implementation of ERAS, which may in part reflect the reduction in VTE risks seen in minimally invasive colectomies. Although ERAS was not directly measured, there is evidence of its impact in our study by the reduction in inpatient length of stay from 12 (IQR 9–18) to 6 (IQR 4–11) days over 2000–2019. Therefore, our study's finding of a year‐on‐year reduction in VTE risk following elective colorectal resection may be associated with the improved perioperative care ERAS patients receive, in addition to more minimally invasive surgery and extended VTE prophylaxis guidelines [61]. Further understanding of the duration of magnitude of VTE risk in this group of patients may allow tailoring of guidance on extended VTE prophylaxis as thresholds for intervention may have changed given that the studies underpinning this guidance included patients mainly undergoing open elective surgery over 15 years ago [6]. However, randomized controlled trial evidence to define this further might not be feasible due to the low absolute risk of postoperative VTE and therefore large overall numbers needed to adequately power a study, suggesting large prospective cohort data might be the more realistic approach.

### Emergency colorectal resection VTE risks

Following emergency surgery, we found an annual reduction in VTE risk following minimally invasive benign resections and a year‐on‐year increase in VTE risk post open malignant colorectal resections. This contrasts with contemporary data reporting no differences in annual VTE risk following emergency colectomy [62]. Although the smaller numbers for emergency minimally invasive procedures mean their trends are underpowered, this again highlights that extended VTE prophylaxis alone might not be able to compensate for other developments in perioperative care.

There was an increase in year‐on‐year VTE risk following open emergency malignant colorectal resections despite guidance supporting extended VTE prophylaxis in this subgroup. However, the ENDORSE study highlighted that emergency compared to elective surgical patients were less likely to receive appropriate VTE prophylaxis [63]. Other possible explanations include the increasingly elderly population undergoing malignant colorectal surgery and increasingly complex Stage IV and metastatic disease resections that may previously not have been attempted, all of which increase VTE risk [54, 64, 65, 66]. Additionally, those undergoing open colectomy are unlikely to be suitable for ERAS programmes. Our study therefore identifies this emergency malignant group as those most in need of appropriate VTE prevention strategies [67]. National initiatives such as the American College of Surgeons National Quality Improvement Program in the US and the National Emergency Laparotomy Audit in the UK could help focus developments in this area and offer a mechanism to record improvements in outcomes over time for this high‐risk group of patients [68, 69].

## CONCLUSION

Over the last 20 years VTE risk following elective colorectal resections has reduced in absolute terms, specifically when compared to the VTE risk following emergency resections. This risk reduction is most probably due to changes in perioperative surgical care. This suggests future focus on improving postoperative emergency colectomy VTE rates is needed with the application of specific advances observed in the elective setting, such as emergency ERAS pathways utilizing minimally invasive techniques where possible.

## AUTHOR CONTRIBUTIONS

All authors meet the conditions for authorship, CRediT. CLL: Conceptualization, Methodology, Software, Validation, Formal analysis, Investigation, Data curation, Writing—Original draft, Writing—Review and editing, Visualization, Project administration. CC: Conceptualization, Methodology, Software, Validation, Resources, Writing—Review and editing, Visualization, Supervision, Project administration. JW: Software, Validation, Resources, Writing—Review and editing, Visualization, Supervision. OP: Validation, Writing—Review and editing, Visualization, Supervision. DH: Conceptualization, Methodology, Software, Validation, Investigation, Resources, Data curation, Writing—Review and editing, Visualization, Supervision, Project administration.

## CONFLICT OF INTEREST

There are no conflicts of interest, use of off‐label or unapproved drugs or products and use of previously copyrighted material to declare.

## ETHICAL APPROVAL AND PATIENT CONSENT

The study was approved by the Independent Scientific Advisory Committee approval board (Protocol 19_180RA3). Data from Hospital Episode Statistics and Clinical Practice Research Datalink if used as instructed, as the authors have, do not require individual specific patient consent and this is implied under the study approval. All material is of the authors’ original creation and there is no clinical trial registration required.

## Supporting information


Appendix S1

## Data Availability

The data that support the findings of this study are available from Clinical Practice Research Datalink (CPRD). Restrictions apply to the availability of these data, which were used under license for this study. Data are available from the author(s) with the permission of Clinical Practice Research Datalink (CPRD).
