# Supplementary material for: Time trends in the incidence rates of venous thromboembolism following colorectal resection by indication and operative technique
Source: Colorectal Dis. 2022 Jul 19;24(11):1405–15. doi: 10.1111/codi.16233 (PMC9796069; doi:10.1111/codi.16233)
Supplement: Supplementary file 1 — Appendix S1 [file CODI-24-1405-s001.docx]

# **ONLINE SUPPLEMENTARY DATA**

**OPCS and ICD codes used to identify colectomy, inflammatory bowel disease and diverticular disease.**

**Supplementary Figure 1. Proportion of (A) elective and (B) emergency colorectal resections by operative technique over time.**

Minimally Invasive Surgery consists of procedures started either by a laparoscopic or robotic technique.

**Supplementary Table 1: Multivariable analysis of 30-day venous thromboembolism risk following colorectal resection per year change**

Minimally Invasive Surgery consists of procedures started either by a laparoscopic or robotic technique.

^a^Adjusted for admission type, surgical indication, age, sex, Charlson score, ethnicity, operative technique and year.

^b^P-value for adjusted model.

This supplementary material has been provided by the authors to give readers additional information about their work.

**OPCS and ICD codes used to identify colectomy, inflammatory bowel disease and diverticular disease**

**Colectomy codes**

H041, H042, H043, H048, H049, H051, H052, H053, H058, H059, H061, H062, H063, H064, H068, H069, H071, H072, H073, H074, H078, H079, H081, H082, H083, H084, H085, H088, H089, H091, H092, H093, H094, H095, H098, H099, H101, H102, H103, H104, H105, H108, H109, H111, H112, H113, H114, H115, H118, H119, H291, H292, H293, H294, H298, H299, H331, H332, H333, H334, H335, H336, H337, H338, H339

**Inflammatory bowel disease codes**

K50, K500, K501, K508, K509, K51, K510, K512, K513, K514, K515, K518, K519, K520, K521, K522, K523, K528, K529

**Diverticular disease codes**

K57, K570, K571, K572, K573, K574, K575, K578, K579

**(A)**

**(B)**

**Supplementary Figure 1. Proportion of (A) elective and (B) emergency colorectal resections by operative technique over time**

Minimally Invasive Surgery consists of procedures started either by a laparoscopic or robotic technique.

| **Per Year Change (2000 - 2019)** | **Incidence Risk (%)** | **Adjusted Incidence Rate Ratio^a^** | | | **Percentage (%) change of Adjusted Model** | | | **P-value^b^** |
| --- | --- | --- | --- | --- | --- | --- | --- | --- |
|  |  |  | **(95%CI)** | | **%** | **(95%CI)** | |  |
| **Elective Benign** |  |  |  |  |  |  |  |  |
| **Open** | 0.57 | **0.98** | (0.96 | 1.00) | **-2.03** | (-4.21 | 0.19) | 0.073 |
| **Minimally Invasive** | 0.27 | **0.93** | (0.90 | 0.97) | **-6.78** | (-10.26 | -3.15) | <0.001 |
| **Elective Malignant** |  |  |  |  |  |  |  |  |
| **Open** | 0.73 | **0.99** | (0.98 | 1.01) | **-0.81** | (-2.48 | 0.88) | 0.344 |
| **Minimally Invasive** | 0.37 | **0.94** | (0.91 | 0.98) | **-5.62** | (-8.84 | -2.27) | 0.001 |
| **Emergency Benign** |  |  |  |  |  |  |  |  |
| **Open** | 0.99 | **1.01** | (0.99 | 1.02) | **0.64** | (-0.99 | 2.29) | 0.446 |
| **Minimally Invasive** | 0.48 | **0.96** | (0.92 | 1.00) | **-4.24** | (-7.88 | -0.45) | 0.029 |
| **Emergency Malignant** |  |  |  |  |  |  |  |  |
| **Open** | 1.43 | **1.02** | (1.00 | 1.04) | **1.89** | (0.05 | 3.77) | 0.044 |
| **Minimally Invasive** | 1.08 | **0.97** | (0.93 | 1.01) | **-3.04** | (-6.78 | 0.84) | 0.123 |

**Supplementary Table 1: Multivariable analysis of 30-day venous thromboembolism risk following colorectal resection per year change**

Minimally Invasive Surgery consists of procedures started either by a laparoscopic or robotic technique.

^a^Adjusted for admission type, surgical indication, age, sex, Charlson score, ethnicity, operative technique and year.

^b^P-value for adjusted model.
